# Sleep Quality Impacts Training Responses and Performance in Elite Swimmers

**DOI:** 10.1002/ejsc.70090

**Published:** 2025-11-25

**Authors:** Emily A. Lundstrom, Mary Jane De Souza, Megan E. Conklin, Nancy I. Williams

**Affiliations:** ^1^ Women's Health and Exercise Laboratory, Department of Kinesiology The Pennsylvania State University University Park Pennsylvania USA; ^2^ Clinical Exercise Research Center, Division of Biokinesiology and Physical Therapy The University of Southern California Los Angeles California USA

**Keywords:** elite athletes, sleep deprivation, sport performance, wearable technology

## Abstract

High‐quality sleep is necessary for optimal health and promoting recovery from training, contributing to sport performance. Research suggests a high prevalence of poor sleep duration and quality in athletes. Reduced sleep duration has been shown to be deleterious to performance, but less is known about sleep quality and its relationship to training responses and performance. In 26 elite male (*n* = 10) and female (*n* = 16) collegiate swimmers, we assessed sleep quality (sleep duration (hrs), sleep debt (hrs), slow‐wave sleep (SWS_hrs_ and SWS%), rapid‐eye movement (REMhrs and REM%)), training measures (strain (AU), average heart rate (HR) (ExHR_avg_) and maximum exercising HR (ExHR_max_)), and swimming performance (200yd time trial swim) during heavy training, preceding championship competition. Collection of sleep data was matched to days of training data collection, and also to the day preceding the performance swim. Pearson correlations were utilized to determine relationships between variables unless sex effects existed in which case linear regression analyses were utilized to control for sex differences in variables. In all swimmers, sleep duration is related to strain (*R* = −0.78; *p* = 0.01), and sleep debt is related to ExHR_avg_ (*R* = 0.53; *p* = 0.005). SWS_hrs_ negatively is related to ExHR_avg_ (*R* = −0.42; *p* = 0.032). Controlling for sex, sleep duration predicted swimming performance (*R* ^2^ = 0.881; *p* < 0.001), swimmers with greater sleep durations exhibited faster swim race times. Similarly, when controlling for sex, SWS% predicted swimming performance (*R* ^2^ = 0.883; *p* < 0.001), swimmers with greater SWS% exhibited faster times. Sleep quality measures were related to training adaptations and swimming performance was predicted by sleep quantity and quality. Athletes should obtain adequate sleep to support recovery and optimize training and performance.

## Highlights

- Sleep quality was related to both training responses and swimming performance in all swimmers
- Swimmers with higher sleep quality exhibited lower average workout strain and heart rates during training
- Swimmers with higher sleep quality exhibited faster swimming performance during the time trial swim.

## 1 Introduction

Competitive collegiate swimming requires not only peak physical conditioning and technical skill, but it also requires sufficient recovery to excel in both training and competition. Among the multifactorial determinants influencing both training responses and sport performance, recovery (often encompassing nutrition, sleep quantity and quality) emerges as a pivotal factor in maximizing athletic potential (Charesta and Grandner 2020; Doherty et al. 2021; Halson 2008). Given the rigorous and frequent training sessions and competitions, elite collegiate endurance athletes often struggle to obtain sufficient sleep for recovery from training (C. D. Mah et al. 2018; Ashby et al. 2024). Insufficient sleep is highly prevalent in elite athletes and is often attributed to the time demands of balancing academic responsibilities with rigorous training schedules at the expense of adequate sleep (C. D. Mah et al. 2018). A study investigating the sleep needs of 175 elite athletes found that, on average, athletes require 8.3 h of sleep to feel rested (Sargent et al. 2021), yet 39.1% of elite collegiate athletes report obtaining fewer than 7 h of sleep during weekdays (C. D. Mah et al. 2018). Insufficient sleep has been demonstrated to relate to reduced health outcomes, such as cognitive decline (Charesta and Grandner 2020), elevated risk for mental health disorders (Charesta and Grandner 2020), impaired immune function (Schmitz et al. 2022), and poor athletic performance (Marshall and Turner 2016). Additionally, factors such as early morning training sessions characteristic to the sport of collegiate swimming have been related to poor sleep (Sargent et al. 2014; Merfeld et al. 2022). Research investigating the sleeping behavior of elite swimmers found that during nights preceding early morning training, swimmers obtained 5.4 h of sleep, whereas they obtained 7.1 h of sleep on night's preceding rest days (Sargent et al. 2014), indicating that early training sessions significantly reduce the amount of sleep obtained by elite swimmers. Poor sleep has been cited as an outcome associated with the exacerbation of overtraining syndrome, often present in overtrained athletes (Lastella et al. 2018), and can result in training maladaptation, including reduced exercise capacity and prolonged recovery time (Belenky et al. 2003; Roberts et al. 2019). Thus, understanding the intricate interrelationships between sleep parameters, training responses, and swimming performance is essential for optimizing training protocols and maximizing competitive performance, particularly among elite collegiate swimmers.

Sleep plays a fundamental role in physiological restoration, cognitive function, and overall well‐being (Venter 2012) and different sleep parameters are associated with different outcomes. Specifically, sleep quantity, commonly measured by total sleep duration, and sleep quality, assessed through parameters such as sleep debt and sleep architecture (including the distribution of slow‐wave sleep (SWS) and rapid eye movement (REM) sleep), are integral components of an athlete's recovery regimen. Adequate sleep duration facilitates tissue repair (Venter 2012; Morrison et al. 2022; H. H. Fullagar, Duffield, et al. 2015; H. H. Fullagar, Skorski, et al. 2015), hormone regulation (Venter 2012; Morrison et al. 2022; H. H. Fullagar, Duffield, et al. 2015; H. H. Fullagar, Skorski, et al. 2015), and neural consolidation (Rasch and Born 2013), whereas disruptions in sleep patterns, such as sleep debt accumulation, can compromise physical and cognitive performance (Roberts et al. 2019; H. H. Fullagar, Duffield, et al. 2015; H. H. Fullagar, Skorski, et al. 2015) and can lead to the development of illness (Medic et al. 2017). In addition to its impact on recovery, sleep quality has been implicated in various aspects of athletic performance, including reaction time (Taheri and Arabameri 2012; C. Mah 2008), decision‐making (Harrison and Horne 2000), and physical endurance (Roberts et al. 2019). SWS, characterized by synchronized neuronal activity and metabolic processes, contributes to physical recovery and healing (Sassin et al. 1969; Léger et al. 2018), whereas REM sleep is associated with cognitive processing and emotional regulation (K. E. Miller and Gehrman 2019).

Training responses, encompassing physiological adaptations to exercise stimuli, provide valuable insights into the effectiveness of training regimens. Objective measures of training and fitness, such as average heart rate, maximal heart rate, and exercise strain (measure of cardiovascular and muscular exertion) from wearable technology may inform an athlete about their readiness to perform. Research examining the impact of training sessions on sleep in elite athletes across seven sports found that the amount of sleep obtained prior to early training sessions was significantly lower compared to rest days, and shorter sleep durations were correlated with pretraining fatigue as measured using a self‐reported fatigue scale (Sargent et al. 2014). Despite the recognized importance of sleep on optimizing training responses and athletic performance, research specifically examining the relationship of sleep with swimming performance, especially within the collegiate elite swimming population, remains limited. Therefore, the purpose of this study was to determine the interrelationships between sleep quality, training metrics, and sport performance.

## 2 Materials and Methods

### 2.1 Study Design

This is a sub‐analysis of a cross‐sectional study in 26 elite collegiate swimmers (10 males and 16 females) examining sleep, training load, training responses, and swimming performance. This study was a part of a broad cross‐sectional investigation in 27 elite male and female swimmers, designed with multiple distinct aims, each addressing separate hypotheses related to within‐day energy balance, metabolic function, utility of the wearable device to assess stress and recovery, and, as presented in this manuscript, sleep, training outcomes, and performance. The current analysis focuses exclusively on sleep, training, and performance data. Further details of the larger study design have been previously published (Lundstrom et al. 2024, 2023). Briefly, data collection occurred over a 6‐week period within the competitive season, during which training load and intensity were at its highest prior to postseason championship competition. Prior to data collection, the study was approved by the University Institutional Review Board, and the informed consent of each participant was obtained. Data collection consisted of assessment of body composition, training volume duration, distance covered during training sessions, training responses (workout strain, average exercise heart rate, and maximal exercise heart rate, energy expenditure) obtained from a wearable device, competitive swimming performance and sleep quantity and quality (sleep_duration_, sleep_debt_, hours, and percentage of), slow‐wave sleep (SWS), and rapid‐eye movement (REM) obtained from a wearable device.

### 2.2 Methodology

#### 2.2.1 Participants

The participants for this study consisted of 27 elite collegiate swimmers (11 males and 16 females) from a University NCAA Division 1 Swim team. The overall study included the recruitment of all eligible and available members of the university's elite NCAA division 1 swim team during the peak‐season data collection window. As this was a cross‐sectional observational study employing a convenience sample in a specific population, a formal a priori sample size calculation was not performed. The final sample comprised the aforementioned 27 swimmers, reflecting the full roster of participants who met the inclusion criteria and consented to participate. We acknowledge the sample's heterogeneity based on sex; the sample size is consistent with prior research in elite athlete populations (C. Mah 2008; Campbell et al. 2021) and provides a valuable dataset for exploratory analyses. Of the 27 total swimmers assessed, one swimmer's data was excluded from the current analysis due to one swimmer entering a taper phase (characterized by significantly reduced training volume) prematurely prior to the completion of data collection; thus, data from 26 swimmers (10 males, 16 females) were used. All participants were free of injury, able to train without modifications, and in good health. More descriptive information about the participants can be found elsewhere (Lundstrom et al. 2024, 2023).

#### 2.2.2 Anthropometrics and Body Composition

Total body mass was measured without heavy clothing or shoes, reporting mass in kilograms to the nearest 0.01 kg, whereas height measurements were recorded in centimeters to the nearest 0.1 cm. Next, using these measurements, weight to height ratio in kg/m^2^ was calculated to obtain the body mass index of each participant.

Dual‐energy X‐ray absorptiometry (DXA) (Hologic Horizon‐W, Model 201,331) was used to assess body composition. DXA scans were performed by an International Society of Clinical Densitometry‐certified technician on the study team. Participants were asked to remove any metal‐containing clothing or jewelry before lying still on a padded bed for approximately 5 minutes while a whole‐body scan was completed to determine fat‐free mass, lean mass, and fat mass.

#### 2.2.3 Training Load and Responses

As detailed elsewhere (Lundstrom et al. 2024; Lundstrom, De Souza et al. 2023), participants were required to attend all scheduled trainings that consisted of coach‐administered workouts inclusive of both in‐water and weightlifting sessions as part of their participation in the study. Using these detailed training schedules, study personnel assessed several variables, including training duration, volume, and activity type in order to determine the overall training load for each week of data collection. Briefly, the training duration in minutes for each participant and the objective training volume as session yardages for each participant were recorded by the study coordinator who attended every training session throughout the data collection period. Measures of training intensity were captured via rating of perceived exertion (RPE) on a scale of 0 to 10, which was obtained for each participant after each training session through the completion of the Borg CR10 scale (Wallace et al. 2009; Borg 1982). Training measurements were collected simultaneously to match sleep measurement, that is, training data were collected for the specific days following each sleep bout.

To capture training responses, this study used the WHOOP (WHOOP, Boston, MA), a wearable device that has demonstrated acceptable validity and reliability against gold‐standard measurements (D. J. Miller et al. 2022) and against validated surrogate heart rate measurement tools used during exercise (Bellenger et al. 2022). Exercise energy expenditure, workout strain, and workout heart rates were measured via the WHOOP, using heart rate as assessed via photoplethysmography, and tri‐axial accelerometry to analyze heart rate during exercise activity and prompting participants to input their type of exercise and confirm its completion. Together, these data points allow the WHOOP to calculate the exercise energy expenditure. Utilizing the exercise energy expenditure and exercise heart rate data, the WHOOP also calculates a strain score, a measure of “cardiovascular load,” on a scale of 0–21 with 0 indicating very low strain and 21 indicating very high strain. Workout strain is quantified by a proprietary algorithm utilizing the duration of time spent in heart rate zones based on an athlete's predicted maximum heart rate (0%–50%, 50%–60%, 60%–70%, 70%–80%, 80%–90%, and 90%–100%). A daily strain score metric is also provided by the wearable as an assessment of the culmination of daily physical activity, but this was not used for the present analysis. Participants were required to wear the WHOOP at all times during the entire study duration. A 24‐h energy expenditure, which included both exercise energy expenditure and nonexercise‐related activity, was also measured by the WHOOP, but was not used for this analysis.

#### 2.2.4 Sleep

Sleep data were captured utilizing the WHOOP via the WHOOP's sleep detection and sleep staging algorithm to produce the various sleep measures (WHOOP Inc., Boston, MA). The WHOOP has been validated against polysomnography for its various sleep measures (D. J. Miller et al. 2020; D. Miller et al. 2021; Berryhill et al. 2020) in active healthy individuals, but not athletes. Although the WHOOP's advanced technology and sleep algorithms allow for the auto‐detection of sleep, as described above, the WHOOP application also allows users to confirm sleep times or manually adjust sleep times to further improve the accuracy of sleep data. The specific sleep variables of interest captured by the WHOOP included total sleep time (hours:minutes), sleep disturbances (number of disturbances accumulated over an entire sleep bout), sleep efficiency ((time asleep/time in bed) * 100), and amount of time (hours:minutes) and percentage of time in each of the four main stages of sleep (wake, light sleep, slow‐wave sleep, and rapid eye movement sleep). In the validation study by D. J. Miller et al. (2022), WHOOP demonstrated moderate agreement with polysomnography for total sleep time, light sleep, deep sleep (SWS), and REM sleep, specifically, correctly identifying 62% and 66% of SWS and REM sleep polysomnography epochs (D. J. Miller et al. 2022), and has provided reliable estimates of trends in sleep stages across multiple nights (D. J. Miller et al. 2022; D. J. Miller et al. 2020; D. Miller et al. 2021; Berryhill et al. 2020). The present study used WHOOP‐derived estimates to evaluate relative differences in sleep characteristics between participants and their relationship to training and performance outcomes, rather than for clinical diagnosis of sleep disorders. Comparisons of sleep assessments of participants were compared to the National Sleep Foundation's recommendations for athletes (Vitale et al. 2019; Doherty et al. 2021; Walsh et al. 2021; Ohayon et al. 2017). For the analysis of sleep and training response data against measures of training responses, sleep data was collected on matched days with training responses and volume data recording. For the analysis of sleep data against swimming performance, sleep data were retrieved for the night preceding the time trial swim, as well as for the 3 days (averaged) leading up to the time trial performance swim.

#### 2.2.5 Performance Time Trial Swim (TT_perf_)

One 200‐yard freestyle time trial was completed during the study's data collection period as a measure of swimming performance (TT_perf_). This time trial, occurring during the team practice time, followed a protocol common to elite swimming participation and was overseen by members of the team's coaching staff as well as certified lifeguards. Prior to the date of the time trial, participants were encouraged to treat the time trial as they would an actual competition and to prepare accordingly. Then, upon arrival to the time trial designated practice, participants were given 30 min to warm up before the time trial began. Participants competed in heats of 6 swimmers each, organized to consist of teammates that they normally compete against, and a cheering section was formed around the pool by the athletes who had already raced or by those not participating in the race to encourage fast swims and to simulate a real race environment. Participants dove off of the starting block and completed a 200‐yard freestyle swim at their maximal exertion level. Both the Colorado Timing system (Colorado Time System; Loveland, CO) and handheld timers were used to time the time trial, which lasted less than 2 minutes for all participants. As aforementioned, sleep data were retrieved for days corresponding to the night(s) prior to the time trial race (the night prior to the race and 3‐day average of sleep prior to the race) for analysis.

#### 2.2.6 Statistical Analyses

Data were analyzed using SPSS Statistical Software (version 26, Chicago, IL). All variables were tested for normality and outliers prior to conducting statistical analyses. First, normality was tested using the Shapiro–Wilk statistic. Then, outliers were located, removed, and Levene's test was utilized to determine the homogeneity of variance. For the analyses, participants were analyzed as a whole group and by sex. Independent *t*‐tests were utilized to determine group differences in variables of interest between sexes. Correlations were calculated using Pearson's correlation analysis to identify relationships of interest. Linear regression analyses were utilized to determine statistically significant predictors of sleep quality, training responses, and time trial performance when sex differences were evident. Data were reported as mean +/− SD, and a *p*‐value of < 0.05 was considered statistically significant.

## 3 Results

### 3.1 Participant Characteristics

A total of 27 NCAA Division I collegiate swimmers (11 males and 16 females) enrolled in this study. One male participant's data were excluded post‐collection due to prematurely tapering before the completion of the data collection period, leaving 26 participants in the final analyses. The racial/ethnic distribution was primarily Caucasian (92%). Further descriptive characteristics are included in Table 1.

**TABLE 1:** Participant characteristics.

	All (*n* = 26)	Male (*n* = 10)	Female (*n* = 16)	
	Mean ± SD	Mean ± SD	Mean ± SD	*p*‐value
Demographics				
Height (cm)	178.7 ± 7.8	186.4 ± 4.9	174.0 ± 4.8	0.001
Weight (kg)	74.1 ± 10.3	83.8 ± 8.6	68.0 ± 5.6	0.001
Age (yr)	19.6 ± 1.1	20.1 ± 1.0	19.3 ± 1.0	0.094
Body mass index (kg/m^2^)	23.1 ± 1.9	24.1 ± 1.9	22.5 ± 1.6	0.024
Fat free mass (kg)	56.5 ± 11.0	68.6 ± 6.7	49.0 ± 4.3	0.001
Fat mass (kg)	16.0 ± 2.8	13.6 ± 2.1	17.5 ± 2.1	0.001

Abbreviations: SD, standard deviation.

### 3.2 Descriptive Energy, Sleep, Training, and Performance Characteristics in all Swimmers

As shown in Table 1, male swimmers had significantly greater height, weight, BMI, fat free mass, lean body mass (Table 1), and faster swim performance (Table 2) than female swimmers (*p* < 0.05). Male swimmers also exhibited significantly greater SWS_hrs_ and SWS% (p < 0.05) (Table 2). No significant sex differences were observed for other sleep measures or training response variables (Table 2). Notably, only 42% of all swimmers (11/26) achieved a sleep duration_hrs_ of at ≥ 7 h across the training days as recommended by the National Sleep Foundation (Ohayon et al. 2017), with lower proportions in females (6/16; 38%) compared to males (5/10; 50%).

**TABLE 2:** Sleep, training, and performance measurements.

	All (*n* = 26)	Male (*n* = 10)	Female (*n* = 16)	
	Mean ± SD	Mean ± SD	Mean ± SD	*p*‐value
Sleep				
Matched to training dates				
Sleep duration (hrs)	6.8 ± 0.9	6.6 ± 1.1	7.0 ± 0.7	0.303
REM duration (hrs)	1.7 ± 0.7	1.9 ± 0.9	1.5 ± 0.5	0.157
SWS duration (hrs)	1.2 ± 0.3	1.4 ± 0.3	1.0 ± 0.3	0.009
Sleep debt (hrs)	1.2 ± 0.5	1.3 ± 0.6	1.2 ± 0.4	0.898
REM percentage	22.1 ± 8.0	23.1 ± 9.8	21.4 ± 6.8	0.599
SWS percentage	18.0 ± 5.6	21.8 ± 4.3	15.7 ± 5.1	0.013
Matched to time trial performance				
Night preceding time trial				
Sleep duration (hrs)	6.8 ± 1.2	6.6 ± 1.2	6.9 ± 1.2	0.630
Sleep debt (hrs)	1.0 ± 0.7	1.1 ± 0.6	0.9 ± 0.7	0.560
REM percentage	20.8 ± 9.8	21.8 ± 9.2	20.3 ± 10.3	0.713
SWS percentage	14.9 ± 4.7	14.5 ± 4.4	15.1 ± 4.9	0.728
Averaged 3 nights preceding time trial				
Sleep duration (hrs)	6.7 ± 0.9	6.5 ± 0.9	6.9 ± 0.9	0.223
Training and performance				
Time trial time (sec)	112.2 ± 6.2	105.1 ± 3.1	116.6 ± 1.9	0.001
Average training volume (yds/d)	6100 ± 950	5900 ± 1000	6250 ± 850	0.687
Workout strain (AU: 0–21)	14.0 ± 1.1	14.0 ± 1.4	14.0 ± 0.9	0.879
Average workout heart rate (bpm)	154 ± 10	154 ± 12	153 ± 8	0.850
Maximum workout heart rate (bpm)	181 ± 6	182 ± 5	180 ± 7	0.501

Abbreviations: REM, rapid eye movement; SD: standard deviation; SWS, slow‐wave sleep.

### 3.3 Relationships Between Sleep and Training Responses

**FIGURE 1:**
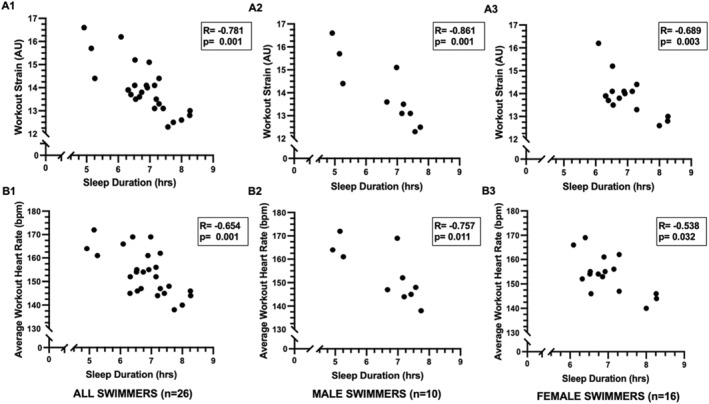
Relationships between sleep duration and training responses in all swimmers. Panels A1–A3 illustrate relationships between sleep duration and workout strain, and panels B1–B3 illustrate relationships between sleep duration and average workout heart rate. Panels A1–B1 include correlations between variables in all swimmers together, panels A2–B2 refer to relationships between variables in the male swimmers only, and panels A3–B3 refer to relationships between variables in female swimmers only.

**FIGURE 2:**
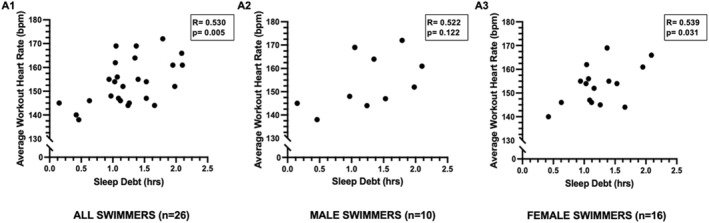
Relationships between sleep debt and average workout heart rate in all swimmers. Panel A1 includes correlations between variables in all swimmers together, panel A2 refers to relationships between variables in the male swimmers only, and panel A3 refers to relationships between variables in female swimmers only.

**FIGURE 3:**
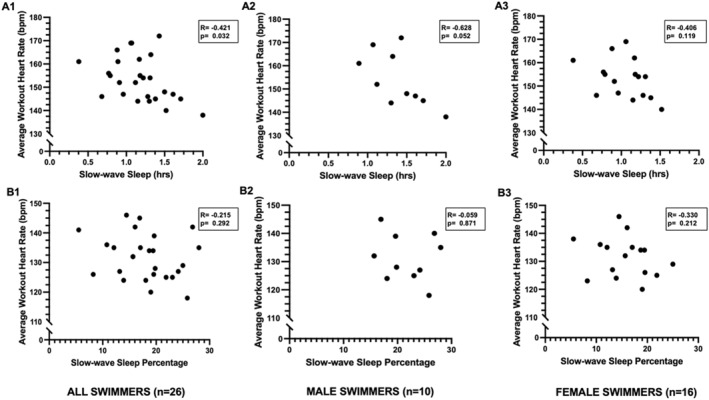
Relationships between slow‐wave sleep measures and average workout heart rate in all swimmers. Panels A1–A3 illustrate relationships between slow‐wave sleep (SWS) hours and average workout heart rate, panels B1–B3 illustrate relationships between slow‐wave sleep percentage and average workout heart rate. Panels A1–B1 include correlations between variables in all swimmers together, panels A2–B2 refer to relationships between variables in the male swimmers only, and panels A3–B3 refer to relationships between variables in female swimmers only.

Relationships between sleep and training responses in all swimmers are displayed in Figures 1, 2, 3, panels A–B. Across all swimmers, shorter sleep duration_hrs_ was negatively correlated with higher workout strain and greater ExHR_avg_ (*p* < 0.05) (Figure 1, panels A1 and B1). Similarly, greater sleep debt was positively correlated with greater ExHR_avg_ (Figure 2A). SWS_hrs_ was inversely related to ExHR_avg_ in all swimmers (Figure 3, panel A1). No significant relationships were evident for SWS% or any REM sleep measurements.

Sex‐specific relationships between sleep and training responses revealed similar patterns and are displayed in Figures 1, 2, 3, panels A2–B2 for male swimmers, and Figures 1, 2, 3 panels A3–B3 for female swimmers. In males, sleep duration_hrs_ was negatively related to workout strain (*r* = −0.861, *p* = 0.001) (Figure 1, panel A2), ExHR_avg_ (*r* = −0.757, *p* = 0.011) (Figure 1, panel B2), and ExHR_max_ (*r* = −0.827, *p* = 0.003), and there was a nonsignificant trend between SWS_hrs_ and ExHR_avg_ (*r* = −0.628, *p* = 0.052) (Figure 3, panel A2). In female swimmers, both reduced sleep duration_hrs_ (Figure 1, panel A3‐B3) and higher sleep debt (Figure 2, panel A3) were correlated to higher workout strain and ExHR_avg_. Additionally in female swimmers, REM_hrs_ was negatively related to ExHR_avg_ (*r* = −0.555, *p* = 0.026). No other associations were found for SWS% or REM% in the sex‐stratified analyses.

### 3.4 The Relationships Between Sleep Quality and Swimming Performance

**FIGURE 4:**
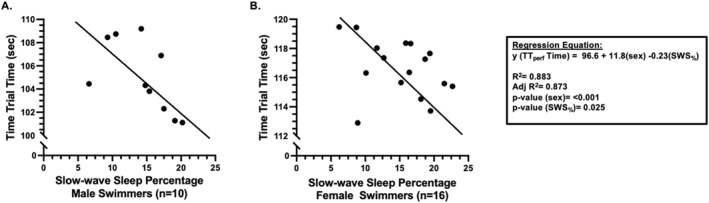
Relationships between sleep quality (slow‐wave sleep) and next morning swimming performance in male and female swimmers. The regression analyses were performed to determine relationships between slow‐wave sleep and next morning swimming performance and to control for the effect of sex. Regression equation, R‐squared and adjusted R‐squared, and *p*‐values are presented in the regression legend. (Panel A) male swimmers; (panel B) female swimmers.

When examining the relationships between sleep quality the night(s) prior to the time trial and swimming performance, there were no significant bivariate relationships evident between sleep measures (sleep duration_hrs_, SWS%, REM%, or sleep debt_hrs_ either the night before the race or when averaging these sleep measures for the 3 days leading up to the race) and swimming performance (TT_perf_) in all swimmers or within sex subgroups. However, when controlling for sex, regression models showed that sleep duration_hrs_ the night preceding the race predicted TT_perf_ (*R* ^2^ = 0.881; *p* < 0.001), whereby swimmers with more hours of sleep swam faster in the time trial. Similarly, when controlling for sex, SWS% the night preceding the race predicted TT_perf_ (*R* ^2^ = 0.883; *p* < 0.001), whereby swimmers with a greater SWS% swam faster in the time trial swim (Figure 4, panels A–B). Regression analyses also revealed that other sleep measures (sleep debt_hrs_, REM_hrs_, and REM% the night before the race, and averaged the 3 nights leading up to the time trial swim) did not predict TT_perf_ in all swimmers when controlling for sex.

## 4 Discussion

This research is the first study to examine the interrelationships between measures of sleep quality and training responses and sleep quality measures on the sport performance of elite swimmers using field‐based methods. Most notably, we found that sleep quality was related to both training responses and swimming performance in all swimmers, whereby swimmers with higher sleep quality exhibited lower average workout strain and heart rates during training and faster swimming performance during the time trial swim. Considered together, these findings suggest that higher sleep quality may result in more favorable training and performance outcomes.

### 4.1 Implications of Insufficient Sleep Duration in a Population of Elite Swimmers

Our study revealed a concerning trend among elite college male and female swimmers, with the majority exhibiting insufficient sleep duration according to the National Sleep Foundation's recommendations for athletes. Specifically, in our cohort of swimmers, only 42% (11/26) of athletes obtained at least 7 h of sleep, as recommended by the National Sleep Foundation. The National Sleep Foundation's recommendation of > 7 h of sleep acknowledges the increased physiological demands placed on athletes engaged in rigorous training and competition (Ohayon et al. 2017). The literature demonstrates that adequate sleep duration is essential for supporting optimal recovery, facilitating physical and cognitive performance, and promoting overall health and well‐being (Halson 2008; Walsh et al. 2021). However, the prevalence of insufficient sleep duration observed in our study highlights a critical area of concern that warrants attention within the elite swimming community.

The implications of insufficient sleep extend beyond mere sleep duration. Disruptions in sleep architecture, including reductions in durations of important sleep stages such as SWS and REM sleep are an outcome of shortened sleep, which may compromise an athlete's ability to recover and adapt to training stimuli (Sassin et al. 1969; Léger et al. 2018; K. E. Miller and Gehrman 2019). This may explain why, in our cohort of male and female swimmers, there was evidence of a significant relationship between SWS_hrs_ (*p* < 0.05) but not SWS% (*p* > 0.05) with ExHR_avg_. Potentially, the benefits of having a greater percentage of sleep spent in the SWS stage may be negated by shorter sleep duration. Moreover, insufficient sleep has been linked to a myriad of negative consequences that can impact training responses and swimming performance (Halson 2008; Schmitz et al. 2022; Marshall and Turner 2016). Sleep deprivation impairs cognitive function, including attention, reaction time, and decision‐making, which are critical for optimizing technique and race strategy in swimming (C. Mah 2008; Taheri and Arabameri 2012). Furthermore, sleep loss compromises immune function, increases susceptibility to illness and injury (Schmitz et al. 2022; Ibarra‐Coronado et al. 2015), and disrupts hormonal balance (Garbarino et al. 2021), all of which can undermine an athlete's capacity to train consistently and perform at their best. Insufficient sleep has been increasingly recognized as a contributing factor to overtraining syndrome in athletes (Campbell et al. 2021; Meeusen et al. 2013; Lastella et al. 2018; Campbell et al. 2021). Athletes experiencing chronic sleep deprivation may exhibit symptoms overlapping with those of overtraining syndrome, including decreased performance (Meeusen et al. 2013; Campbell et al. 2021; Hausswirth et al. 2014), increased susceptibility to illness (Meeusen et al. 2013; Hausswirth et al. 2014), mood disturbances (Campbell et al. 2021), and altered hormonal profiles (Meeusen et al. 2013; Garbarino et al. 2021). Specifically, one study found that the male triathletes participating in a high intensity training program, those with declines in performance, declines in VO_2_ max, and high perceived fatigue (markers of overtraining syndrome) exhibited decreased sleep duration, decreased sleep efficiency, and higher prevalence of upper respiratory tract infections compared to the nonoverreached group (Hausswirth et al. 2014), providing evidence of a connection between sleep and overtraining indicators (Hausswirth et al. 2014). Thus, optimizing sleep duration and quality is imperative in mitigating the risk of overtraining syndrome and maintaining athletes' overall health and performance.

### 4.2 Relationship Between Sleep and Training Responses

The present study demonstrated that greater sleep durations and less sleep debt, a variable that measures insufficient sleep duration and accumulates as individuals fail to obtain the amount of sleep their body demands (WHOOP Inc., Boston, MA), were associated with more favorable training responses in all swimmers. During sleep, the body undergoes systemic processes such as tissue repair, muscle protein synthesis, and hormone regulation, all of which contribute to enhanced muscular function and cardiovascular efficiency (Lamon et al. 2021; Kim et al. 2015; H. H. Fullagar, Duffield, et al. 2015; H. H. Fullagar, Skorski, et al. 2015). Importantly, sleep duration has been linked to restoration of autonomic nervous system balance, with longer sleep durations favoring parasympathetic dominance and promoting cardiovascular stability (Takase et al. 2004; Dettoni et al. 2012), translating to lower resting heart rates and reduced exercise‐induced tachycardia (Baharav et al. 1995; Zhong et al. 2005). One study investigating the effects of increasing sleep duration on athletic performance in collegiate swimmers found faster reaction times off the blocks, improved turn times, improved 15‐m sprint times, and increased kick strokes after increasing sleep duration to at least 10 h per night (C. Mah 2008).

Furthermore, longer sleep durations afford athletes the opportunity for increased SWS. Enhanced SWS duration has been correlated with improved physical performance and reduced perception of exertion during exercise, potentially contributing to the observed reductions in workout strain among elite swimmers with longer sleep durations (Shapiro et al. 1984; Van Helder and Radomski 1989). The present study also found that greater SWS_hrs_ is related to lower ExHR_avg_. The literature supports that SWS carries many physiological benefits, particularly when it comes to growth, healing, and recovery (Sassin et al. 1969; Léger et al. 2018; Schmitz et al. 2022). Such benefits of SWS include maintenance of the diurnal rhythm of human growth hormone that supports the building and repairing of bone and muscle tissue observed during SWS (Sassin et al. 1969; Gronfier et al. 1996). One study investigating the relationship between human growth hormone secretion and slow‐wave EEG activity reported that growth hormone secretory pulses were concurrent with the SWS peaks during the sleep cycle (Gronfier et al. 1996). Furthermore, Gronfier et al. found a positive correlation between the amount of secreted human growth hormone and SWS wave activity (Gronfier et al. 1996). High energy expenditure throughout the day, as is common among elite endurance athletes, increases human growth hormone secretion (Kanaley et al. 1997); however, it has been reported that when athletes obtain less SWS_hrs_, the levels of human growth hormone drop significantly (Kato et al. 2002). Such studies demonstrate that the body is equipped to alter hormone secretion in response to exercise to allow for necessary physiological growth and repair, but insufficient sleep interferes, impairing recovery, which may impact training responses and eventually sport performance (Charesta and Grandner 2020; Venter 2012; Belenky et al. 2003). Without experiencing the proper recovery that adequate SWS promotes, athletes are missing opportunities to gain muscle and bone strength while putting themselves at the increased risk of injury (Milewski et al. 2014), both serving as additional potential avenues for insufficient SWS to impair training response and athletic performance. Altogether, understanding of the role of SWS in facilitating proper recovery provides a strong explanation for the findings in the present study that more SWS was associated with more optimal training responses and faster time trial swim times.

### 4.3 Relationship Between Sleep and Swimming Performance

We found that when controlling for sex, sleep duration_hrs_ the night preceding the race predicted TT_perf,_ whereby swimmers with more hours of sleep swam faster in the time trial. This finding is in agreement with the literature that longer sleep duration relates to improved athletic performance (Schwartz and Simon 2015; C. D. Mah et al. 2011). Investigation of sleep extension on sport performance in collegiate tennis players demonstrated that an increase in sleep duration of 2 hours per night significantly increased serving accuracy (Schwartz and Simon 2015). Similarly, in collegiate basketball players, several measures of performance, including sprint time, shooting accuracy, and free throw percentage improved the following sleep extension (C. D. Mah et al. 2011). Considered together, these studies highlight the role of adequate sleep duration in optimizing various measures of sport performance.

In our cohort of swimmers, we did not find a relationship between sleep debt_hrs_ and TT_perf_. Similar results were demonstrated in elite soccer players, where athletes randomly assigned to complete sleep hygiene strategies had significantly greater sleep duration but no significant difference in any physical performance markers compared to the control (H. Fullagar et al. 2016). Potentially, factors such as arousal, motivation, and competitive drive could have negated the effect of sleep debt on sport performance. Another potential explanation is that increased sleep debt may increase sympathetic capacity during sprint performance, confounding the relationship between sleep debt and sport performance (H. Fullagar et al. 2016).

Higher quality sleep was also associated with faster swimming performance in the present study. Specifically, when controlling for sex, SWS% the night preceding the race predicted TT_perf,_ whereby swimmers with a greater SWS% swam faster in the time trial. A potential explanation for the relationship between sleep duration and SWS is that when athletes chronically demonstrate a short sleep duration, reflected through accumulating a large sleep debt, they spend less total time in each sleep stage, including SWS (Venter 2012; Ohayon et al. 2017). SWS and its physiological benefits are vital to training responses and athletic performance (Léger et al. 2018, Vitale et al. 2019; Sassin et al. 1969, Venter 2012), largely due to the interrelationship between SWS and human growth hormone as previously described (Kanaley et al. 1997; Kato et al. 2002) and reductions in SWS time resulting from short sleep duration provide a possible explanation for the finding that longer sleep duration is related to improved training responses and sport performance.

### 4.4 Limitations

Our study has limitations. First, we used a cross‐sectional design. Therefore, it is not possible to draw conclusions about causality; instead, only associations can be analyzed. Additionally, given the elite caliber of swimmers enrolled in the study and that data were collected only during the intensified train period of the athletes' season, the applicability of the results is limited to athlete type, competition level, and season timepoint. Also, while the sample size of 26 athletes used for the analyses is relatively small, it reflects a sample of convenience and approximately half of the total number of members of a Division I elite swim team—a population that is both difficult to access and inherently limited in size. Although this limits generalizability and may reduce statistical power for detecting smaller effects, the present study still provides valuable insights into sleep and performance patterns in a real‐world elite sport context. Future research, particularly controlled trials with larger and multi‐team cohorts, is warranted to confirm and extend these findings. Furthermore, the significant sex differences between several of the variables of interest (i.e., sport performance TT_perf_, SWS_hrs_ and SWS%) required certain statistical analyses to be performed separately by sex (specifically, correlation analyses), lowering the effective sample size for several of the analyses and, thus, further lowering the statistic power of the result. Aiding the statistical power of the results, however, is the fact that all of the participants that enrolled in the study completed all study procedures and demonstrated good adherence to the protocol, which is a notable strength of this study.

Regarding data collection, another limitation of this study was that in‐laboratory polysomnography, the gold standard for measuring sleep due to its ability to collect sleep quantity and quality data with more accuracy than any other assessment tool (Landry et al. 2015), was not used to collect sleep data. Instead, the WHOOP Performance Optimization system (WHOOP Inc., Boston, MA) was utilized. Although not the gold standard, the WHOOP has been validated against polysomnography for its various sleep measures (D. J. Miller et al. 2020; D. Miller et al. 2021; Berryhill et al. 2020). However, it is important to note that the WHOOP sleep validation studies were not performed in an athletic population (D. J. Miller et al. 2020; D. Miller et al. 2021; Berryhill et al. 2020), and to the authors' knowledge, there has been no validation of the WHOOP “strain” metric.

### 4.5 Future Directions

Although this study contributes novel findings, particularly finding that better sleep quality is associated with more favorable training response and swimming performance in our population of elite swimmers, more research is needed to clarify the effect of sleep quality measures on athletic performance. Particularly, randomized control trials should be conducted to explore the casual relationship between these variables. Additionally, future research on the effects of sleep quality measures on sport performance may focus on examining these relationships across sports throughout different points of the training season and across competitive levels to extend the results of these studies beyond elite collegiate swimmers. Furthermore, although in‐laboratory polysomnography is the gold standard for measuring sleep due to its ability to collect sleep quantity and quality data with the upmost accuracy (Landry et al. 2015), the accessibility, convenience, and growing validation of wearable technology devices like the WHOOP Performance Optimization system (WHOOP Inc., Boston, MA) against the gold standard make wearable technology the future of sleep research. As such, WHOOP sleep validation studies conducted specifically in athletic populations would be helpful in increasing the strength of scientific rigor and applicability of future sleep studies to athlete populations.

## 5 Conclusion

Our findings highlight the prevalence of insufficient sleep duration among elite college male and female swimmers and emphasize the importance of addressing sleep habits to optimize training and performance outcomes. Similarly, sleep quantity and quality were related to swim training responses in elite swimmers. Similarly, performance was predicted by sleep quantity and quality. To avoid negative training and performance consequences of poor sleep quality, athletes should get adequate sleep to support and optimize training and performance. By prioritizing sleep hygiene practices and implementing targeted interventions to improve sleep duration and quality, athletes can enhance recovery, maximize training adaptations, and optimize competitive performance in the demanding sport of swimming.

The implications of our results on training and performance stemming from insufficient sleep highlight the need for comprehensive sleep education and intervention strategies within the sport of elite swimming. Coaches and trainers should prioritize sleep education and emphasize adequate sleep for athletes as an integral component of athletic development and incorporate strategies to promote healthy sleep habits and optimize sleep environment. The National Sleep Foundation recommends emphasis around establishing consistent bedtime routines (Sletten et al. 2023), minimizing exposure to electronic devices before bedtime (Gradisar et al. 2013), and creating conducive sleep environments conducive to quality rest (Sletten et al. 2023; Gradisar et al. 2013). Addressing sleep habits and optimizing sleep hygiene practices among competitive swimmers may mitigate the potential consequences on sport performance and well‐being.

## Funding

The authors have nothing to report.

## Conflicts of Interest

The authors declare no conflicts of interest.

## Data Availability

Data will be made available upon reasonable request to the corresponding author.
